# Medical Virology of Hepatitis B: how it began and where we are now

**DOI:** 10.1186/1743-422X-10-239

**Published:** 2013-07-20

**Authors:** Wolfram H Gerlich

**Affiliations:** 1 Institute for Medical Virology, National Reference Center for Hepatitis B and D, Justus Liebig University Giessen, Schubert Str. 81, 35392, Giessen, Germany

**Keywords:** Hepatitis B virus, Hepadnavirus, HBsAg, Anti-HBc, Anti-HBs, Vaccine, HCC

## Abstract

Infection with hepatitis B virus (HBV) may lead to acute or chronic hepatitis. HBV infections were previously much more frequent but there are still 240 million chronic HBV carriers today and ca. 620,000 die per year from the late sequelae liver cirrhosis or hepatocellular carcinoma. Hepatitis B was recognized as a disease in ancient times, but its etiologic agent was only recently identified. The first clue in unraveling this mystery was the discovery of an enigmatic serum protein named Australia antigen 50 years ago by Baruch Blumberg. Some years later this was recognized to be the HBV surface antigen (HBsAg). Detection of HBsAg allowed for the first time screening of inapparently infected blood donors for a dangerous pathogen. The need to diagnose clinically silent HBV infections was a strong driving force in the development of modern virus diagnostics. HBsAg was the first infection marker to be assayed with a highly sensitive radio immune assay. HBV itself was among the first viruses to be detected by assay of its DNA genome and IgM antibodies against the HBV core antigen were the first to be selectively detected by the anti-μ capture assay. The cloning and sequencing of the HBV genome in 1978 paved the way to understand the viral life cycle, and allowed development of efficient vaccines and drugs. Today’s hepatitis B vaccine was the first vaccine produced by gene technology. Among the problems that still remain today are the inability to achieve a complete cure of chronic HBV infections, the recognition of occult HBV infections, their potential reactivation and the incomplete protection against escape mutants and heterologous HBV genotypes by HBV vaccines.

## Introductory remark

The history of modern research on viral hepatitis began in the year 1963, when Nobel Prize winner Baruch S. Blumberg (1925–2011) reported for the first time publicly on the discovery of a new antigen named Australia antigen (AuAg) (reference [1], page 82). In the years following, AuAg would become the first specific marker of viral hepatitis. Thereafter, viral hepatitis type B became a driving force for the development of modern virus diagnostics and vaccines. This article will recapitulate the major advances in the field of hepatitis B research throughout the last 50 years and point out some perspectives for future research.

## Early methods of virology

### Virus detection

In the 1960s, virology was still a young science, primarily dedicated to basic research. Its clinical relevance was limited. While scientists had been successful in propagating viruses of many major infections in cell cultures, these techniques were suboptimal for diagnosis of most viral diseases. It usually took a long time for the cytopathic effect of virus growth to become evident in cell culture, and many virus strains were only able to grow in the artificial host cell systems after a protracted adaptation. In addition, there were many viruses that did not generate any cytopathic effect, and could therefore not be recognized even if virus replication did occur. Occasionally, observing the viral material with an electron microscope or using certain biological methods, e.g. hemagglutination, were helpful in such cases. These methods of virus detection enabled the development of vaccines, but for clinical diagnostics on an individual basis they were too time-consuming, too difficult, or unsuitable.

### Detection of antiviral antibodies

As an alternative approach, detection of antibodies which the patients had produced against the antigens of the disease-causing agents was adopted as a diagnostic method. However, this approach was problematic as it depended upon detection of the reaction of the patient’s antibodies with the viral antigen. The method most often used was the quite difficult complement fixation reaction (CFR), which was originally developed for diagnosing syphilis. In addition to the human patient serum, and the viral antigen (e.g. from infected chicken embryos or tissue cultures), one needs sheep erythrocytes as indicator cells, rabbit antibodies against the sheep erythrocytes for generating an immune complex on the erythrocyte membrane and, finally, complement (usually from guinea pigs) for the CFR. Complement is a multi-protein complex in animal sera that binds to immune complexes. When assembled and activated on cell membranes, holes are punched in the cells by the complement, which leads to lysis of the cells. If the patient serum does not contain antibodies, the complement lyses the erythrocytes, and the non-transparent red reaction mix becomes a transparent red. If immune complexes were formed previously in the mix of patient serum and viral antigen, these bind the complement away, such that it no longer can lyse the erythrocytes. CFR requires four complex biological component mixtures from four different animal species and these mixtures must all be standardized quantitatively by the individual lab.

One qualitative CFR result was usually not sufficient, since the antibodies detected could have come from a previous, unrecognized infection and not from the current illness. For diagnosis of highly prevalent acute infections one had to demonstrate an increase in antibody titers. To achieve this a first sample was required, which should have been taken as soon as possible after the start of the illness, followed by a second serum sample from the patient, at least one week later that had to contain a significantly higher amount of antibodies against the corresponding virus. Quantification of CFRs and similar biological reactions was only possible by diluting patient sera serially in steps of two and determining the highest dilution that had just given a positive result. Only a titer increase of at least a factor of 4 could be rated as significant. Early virus diagnostics therefore had to use complicated methods which are almost forgotten today. However, the virologists of the 1960’s would have been happy to have these methods available for diagnosing viral hepatitis.

### Early recognition of viral hepatitis

The infectious nature of the disease had already been recognized in the early days of medical microbiology, in 1885 by Lürmann during an “icterus epidemic” which occurred after a small pox vaccination campaign. The vaccine had been made from human “lymph” (probably obtained from the vaccine-induced lesions in other vaccinated persons) [2]. Furthermore, addition of human serum to vaccines was not unusual at that time and e.g. considered necessary to stabilize yellow fever vaccine. Several outbreaks of hepatitis were observed in recipients of yellow fever vaccine [3], the largest in 1942 among U.S. American Army personnel with 50,000 clinical cases. Retrospective analysis showed that the outbreak was caused by hepatitis B virus (HBV) with probably 280,000 additional unrecognized infections [4]. Epidemiological observations in the first half of the last century pointed to at least two types of sub-cellular pathogens: Type A mainly affected children, was spread at often epidemic levels via food or drinking water contaminated with feces and was never chronic. Type B was often transmitted through medical interventions in which human blood or serum was intentionally or, due to a lack of hygiene, accidentally injected or inoculated from one person to the next [3]. The most serious problem of blood transfusion was that even a blood donor in apparently perfect health, who had never had jaundice, could transmit the disease to the recipient and that the recipients often developed severe acute or chronic hepatitis.

### Human experimentation

All attempts to identify the pathogen were unsuccessful for more than eighty years. The problem with viral hepatitis was so big that there was even human experimentation done at several places before and during World War II [3], and among so-called volunteer participants who were, in fact, prisoners in the 1950’s in the USA [5]. Even more ethically problematic were the studies in a large number of mentally handicapped children carried out until 1970. Using targeted infection, New York pediatrician Saul Krugman showed 1967 that there were indeed two separate pathogens causing hepatitis [6]. However, even this experimentation - unimaginable by today’s ethical standards - did not lead to the urgently needed break-through.

## Discovery of Australia antigen

### Search for genetic markers

The first hint came from an unexpected source. American physician and geneticist Baruch Blumberg wanted to study genetic markers for susceptibility to certain illnesses, especially cancer, and had gathered serum samples world-wide from a wide variety of ethnic sources in the 1950′s and 60′s. The possibilities for recognizing genetic differences with laboratory methods were very limited at that time. Blumberg used an immunological approach. He postulated that people, who had received blood products from a large number of donors, e.g. due to hemophilia, would have developed antibodies against “polymorphic” serum proteins. These are proteins that show small genetic differences from person to person in the amino acid sequence, and may be recognized as foreign to the body of the recipient after a transfusion. Blumberg’s co-worker, Harvey Alter, did indeed discover a new antigen in several samples of the huge serum collection, quite often in Australian aborigines, for whom the Australia Antigen (AuAg) was named.

### Relation to hepatitis B

At first, Blumberg believed that AuAg was indeed a polymorphic serum protein like the lipoprotein antigen which he had discovered before. But soon the evidence accumulated that it might have something to do with hepatitis, which Blumberg first revealed in a publication in 1967 [1], page 100. Parallel to that, Alfred Prince (New York Blood Center) was specifically looking for a “serum hepatitis (SH) antigen” in the blood of hepatitis B patients and reported on it in 1968, but soon he realized that it was identical to AuAg [7]. Subsequently, various groups confirmed that Au/SH-Ag was actually a marker for acute or chronic hepatitis B and that there were apparently healthy Au/SHAg carriers.

### Screening for infectious blood donors

A way had been found to recognize infectious blood donations and to sort them out. Blumberg used the agar gel double diffusion developed by Ouchterlony in the discovery of AuAg. Compared to the biochemical methods of protein determination, the Ouchterlony method was fairly sensitive and specific and compared to CFR, it was also technically simple. For infection diagnostics, however, the test was in most cases not sensitive enough. Although blood donation facilities were soon examining all of their donors for AuAg, many post-transfusion hepatitis B infections continued to appear. Thus, the agar gel diffusion and similar methods were only a temporary solution, and the breakthrough came shortly thereafter.

## Development of the first radioimmunoassay (RIA) for an infectious agent

### Chemical labeling of antibodies

In 1972, a team including biochemists Lacy Overby, Ghung-Mei Ling and Richard Decker at Abbott Laboratories (North Chicago) developed a new testing principle for highly-sensitive detection of antigens or antibodies, the solid-phase sandwich radioimmunoassay named *Ausria 125*[8]. The test combined the specificity of the biological antigen-antibody interaction with the high sensitivity of modern physicochemical analytical methods. Proteins can be covalently coupled with many physically detectable components. Even before 1972, it was possible to couple e.g. fluorescent molecules to antibodies. Antibodies visible under UV light were specifically used in the recognition of viral and other causal agents in microscopic tissue preparations (immunofluorescence), and – conversely – fluorescently marked animal antibodies against human antibodies could be used to determine whether a patient serum contained antibodies against a pathogen-specific antibody under the microscope. For viral hepatitis, however, there were no susceptible tissue cultures. One part of this new test took up the newly developed technique of labeling antigens or antibodies with radioactive substances such as iodine-125 which had already facilitated e.g. the detection of insulin and other small molecules by biophysical techniques.

### Solid phase sandwich assay

The completely new aspect was the binding of one component in the test system, here the unlabeled AuAg antibody, by simple adsorption to a surface (solid-phase), and then allowing the analyte in the patient serum, here the AuAg, to specifically bind to that coated surface. The specific binding of the AuAg to the solid phase was thereafter detected by the binding of the Iodine-125 labeled AuAg antibody. Since one AuAg particle contains about 100 binding sites for the antibodies, it was possible for the AuAg to bind first to the solid phase and, in a second step, many molecules of radioactively labeled AuAg antibodies. Non-bound antibodies just had to be thoroughly washed away, and the amount of radioactive iodine 125 on the solid phase (at the time, a plastic bead) was measured. Since radioactivity can be detected with very high sensitivity, the process opened new dimensions in detection sensitivity that could be pushed from several micrograms/ml to a few nanograms/ml AuAg. In addition, the subjective visual reading of a reaction result was replaced by an objective, quantitative measurement method. The new test was a breakthrough for the screening of blood donors and for the diagnosis of viral hepatitis and was very rapidly introduced into clinical practice.

## Discovery of the hepatitis B virus particle (HBV)

### AuAg - an infection marker

In 1970, it was unclear exactly what AuAg was. Was it a host protein formed as a reaction to a pathogen causing hepatitis, was it a component of the pathogen, or was it possibly the pathogen itself? These questions were not simple to answer, since HBV or AuAg could still not be propagated in cell cultures or laboratory animals. In 1971, a first clue was the discovery by Le Bouvier of the AuAg subtypes with antigen determinants *d* or *y*, which appear mutually exclusive in addition to the common determinant *a*[9]. Soon after, Bancroft identified the mutually exclusive determinants *w* or *r*[10]. Within an infection chain, the AuAg subtype always remained the same, which was an argument against the host protein hypothesis and for the pathogen theory.

### Unusual structure of Australia antigen

Under the electron microscope (EM), purified AuAg appeared as small round particles which, unlike viruses, were of variable sizes between 17–25 nm [1], page 108. Most importantly, Blumberg and his team came to the conclusion that these particles did not contain any nucleic acid. Their experimental approach was a bit questionable from today’s ethical standards, but not comparable to Krugman’s experiments. They injected 1 millicurie radioactive ^32^P-phosphate as a biochemical precursor for newly synthesized DNA to a terminally ill HBsAg carrier, obtained 250 ml HBsAg containing plasma after one day, purified the HBsAg and followed the distribution of ^32^P in a density gradient. The purified HBsAg particles contained a small amount of ^32^P, but with phenol extraction it did not remain in the aqueous phase as did nucleic acids but went into the protein/lipid containing phenol phase [11]. In retrospect it is apparent that the conclusion was correct but the experiment was inconclusive, because the nucleic acid of HBV is covalently linked to a protein and is therefore extracted to the phenol phase. Independently, the author showed that the ultraviolet-spectrum of purified HBsAg was that of pure protein and not that of a nucleoprotein [12]. These findings were incompatible with the nature of viruses. Blumberg was at that time, however, so convinced that AuAg was an infectious hepatitis pathogen that he postulated for a while a novel nucleic acid-free infection principle that he named *ICRON* after the Institute for Cancer Research in Philadelphia where he worked at that time [1], page 109. The hypothesis was already formulated by J. S. Griffith [13] that the pathogen of a spongiform encephalopathy (scrapie in sheep) was infectious but would not have any nucleic acid, which Stanley Prusiner was later able to prove with his *prion* theory.

### Discovery of the Dane particle

**Figure 1:**
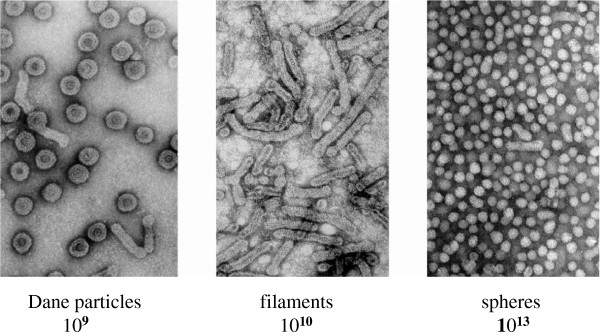
Electron microscopy images (negative staining) and approximate numbers of HBV associated particles in 1 ml of the serum from a highly viremic chronically infected HBV carrier.

**Figure 2:**
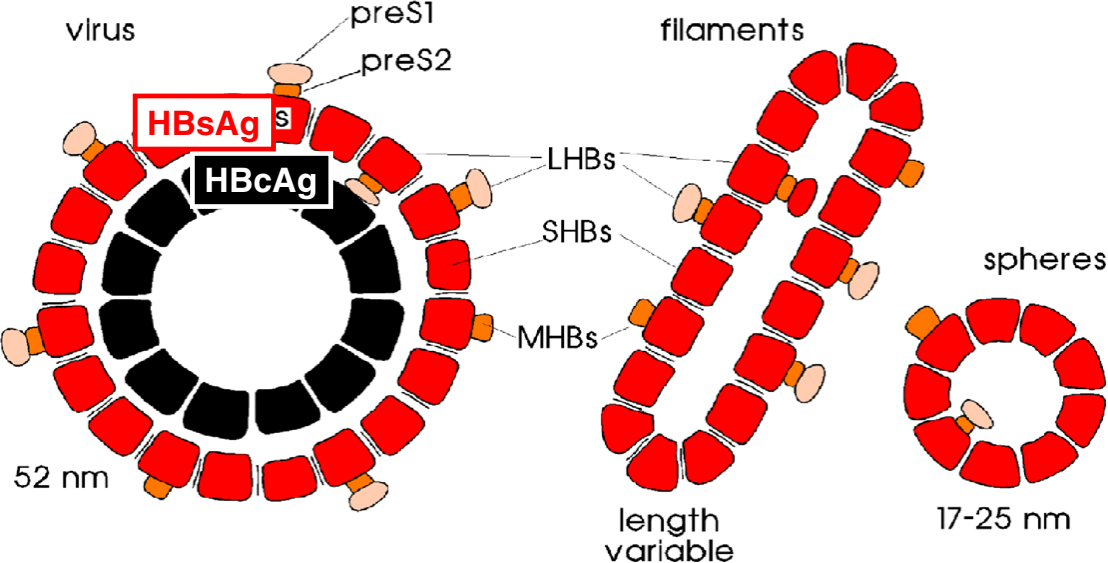
**Model of the hepatitis B virus (Dane particle) and the filamentous or spherical HBsAg particles.** Dane described the virus as 42 nm particle but in the negative staining the outer preS1 and preS2 domains were not visible. 52 nm is the hydrodynamic diameter (Ch. Schüttler and W. Gerlich, unpublished) and also measured by cryo-EM as the outer diameter [16], suppl. Figure 2. HBsAg protein comes in three forms: large (L-) HBs protein with the preS1, preS2 and S domain, middle protein (M-) without preS1 and SHBs without preS1 and preS2.

AuAg, however, was not a prion-like agent. While inspecting AuAg immune complexes under the EM in 1970, David S. Dane (London) discovered that AuAg appeared not only on the small pleomorphic particles, but also on larger, virus-like objects 42 nm in size with a clearly visible inner core [14]. Shortly thereafter, in 1971, his British colleague June Almeida was able to release the core particles from the so-called “Dane particles” by treatment with mild detergent, and showed by immune EM that hepatitis B (HB) patients formed antibodies (anti-HBc) against this core antigen (HBcAg) [15]. This strongly suggested that the Dane particles were the actual virus causing hepatitis B. AuAg was obviously the surface antigen of the virus envelope, and was named HBsAg (s for surface) thereafter. The infected hepatocyte forms the HBsAg protein in large surplus and secretes it in addition to the complete virus as round or filamentous noninfectious particles of about 20 nm in diameter into the blood leading to an approximately three-thousand fold excess of these subviral particles (Figures 1 and 2). This was the reason that the Dane particles could not be recognized in AuAg preparations purified by ultracentrifugation or size chromatography.

### Discovery of HBV DNA

**Figure 3:**
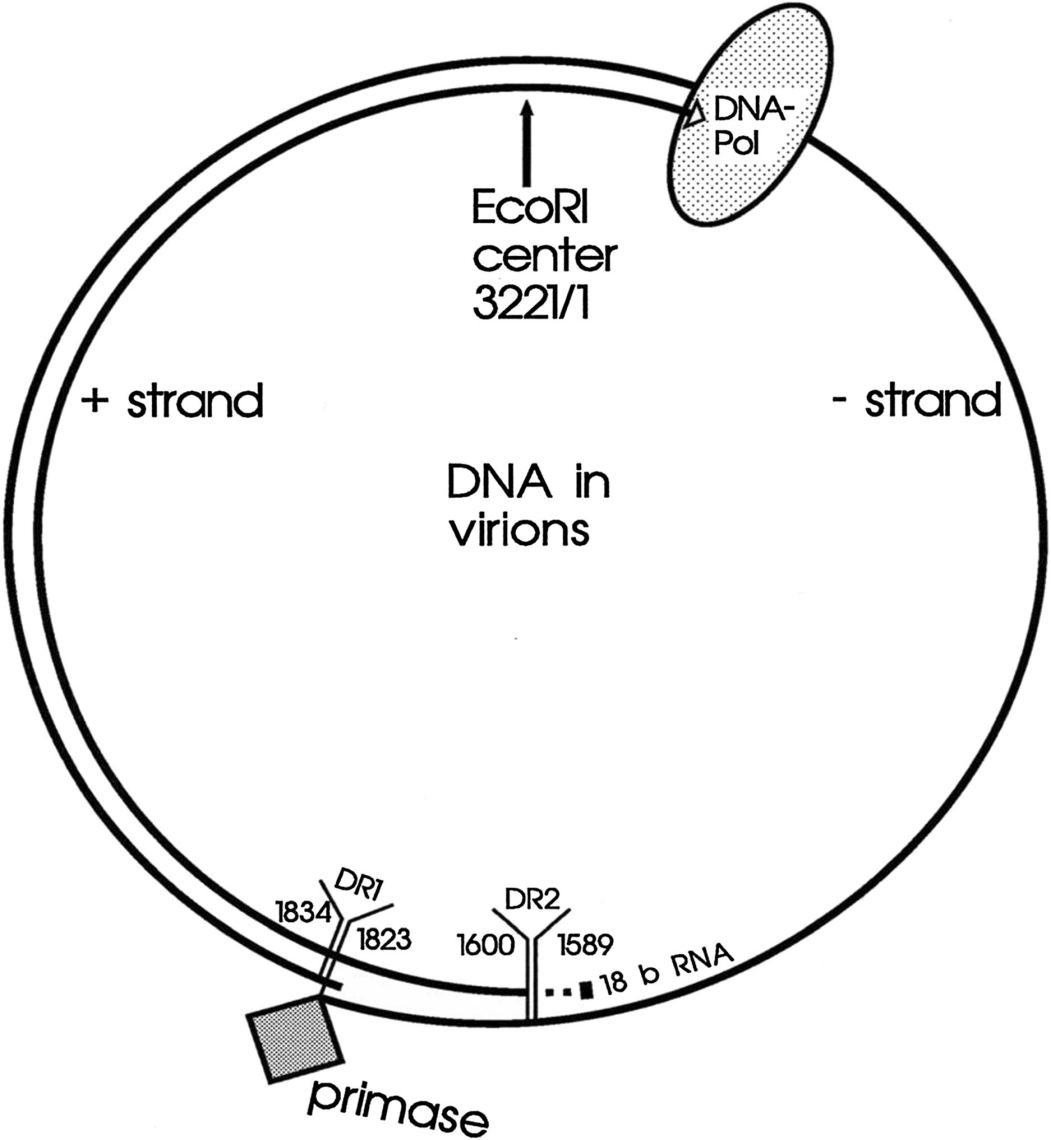
**Biochemical structure of HBV DNA.** The molecule is open circular. The minus-strand has full length within the core particle and even a redundancy of 9 bases at the ends around the nick. The plus-strand is incomplete leaving a large single-stranded gap. The 5′ end of the minus-strand is covalently linked to the primase domain of the DNA polymerase which is present with its active center of the reverse transcriptase domain at the 3′ end of the plus-strand. The 5′ end of the plus-strand contains still its primer which is - in this case- derived from the 18 capped 5′ terminal bases of the degraded pregenomic HBV mRNA.

Since the HBV still could not be grown in cell cultures or in practical laboratory animals, and patient sera contained no more than few nanograms (ng) of Dane particles/ml at most, a direct biochemical detection of the nucleic acid within HBV was not possible at that time. Several researchers tried to identify the HBV genome by indirect methods. Shalom Hirschman (New York) claimed in 1971 that purified AuAg preparations would contain a reverse transcriptase like retroviruses, i.e. a DNA polymerase which accepts *exogenously* added *RNA* templates [17]. Although this exciting publication could not be confirmed, it opened the route to the detection of the HBV genome. When trying to reproduce that report, in 1973 William S. Robinson (Stanford, California) was indeed able to detect an *endogenous DNA* polymerase activity within HBV [18] and, in 1974, he identified the product of that activity itself – the viral DNA [19]. One feature of HBV helped with the recognition of its DNA even without the techniques of molecular biology available today. The DNA within the core particle is, in principle, double-stranded. However, the viral DNA polymerase does not finish synthesizing one strand before the virus is released into the bloodstream. There, it lacks the nucleotide triphosphates which are the building blocks for further DNA synthesis, such that there remains a single-stranded gap in the viral DNA (Figure 3). If one provides those building blocks in the test tube, the synthesis restarts in the form of an *endogenous* DNA polymerase reaction (i.e. without externally added nucleic acid template), and if the nucleotide triphosphates are radioactively labeled this process can not only be detected, but the viral DNA could also be characterized as a small open circular DNA with ca. 3200 bases. In view of these findings, it was generally believed that Dane particles were viruses, but Robinson himself insisted that it would be first necessary to prove that the DNA would encode the viral proteins and infectious HBV. He planned to clone the small amounts of available viral DNA in *E. coli* with the then-newly developed methods in molecular biology. But he was not allowed to do this because of safety concerns about genetically altered organisms which had been raised at the famous conference of Asilomar [20].

### Cloning of HBV DNA

**Figure 4:**
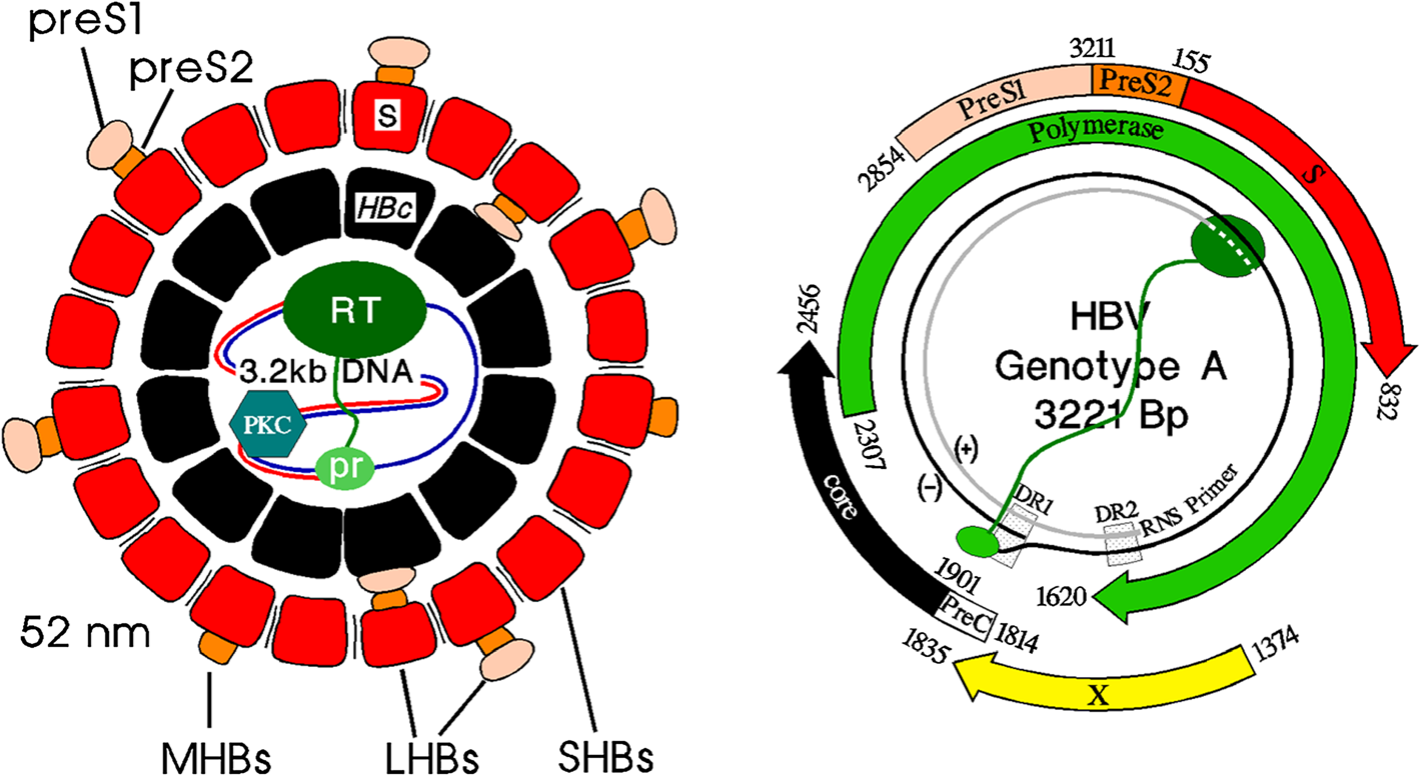
**Structural components of HBV (left) and open reading frames (ORF) for encoding proteins in the covalently closed form of HBV DNA.** The HBV core contains besides the HBV genome the HBV polymerase with the primase (pr) and the reverse transcriptase (RT) domain and the cellular protein kinase C alpha (PKC) [24]. Note the two start codons in the PreC/core ORF and the three start codons in the HBs ORF.

Some years later, around 1978, cloning and sequencing of the HBV DNA was reported almost simultaneously by three other pioneers in molecular biology and their teams who had a biosafety laboratory: Pierre Tiollais (Paris) [21], William Rutter (San Francisco) [22] and Kenneth Murray (Edinburgh, 1930–2013) [23]. The cloned DNA was shown to be indeed circular and to encode the genes for HBsAg, HBcAg, the putative endogenous DNA polymerase and an unexpected X gene (Figure 4). Thus, the cloned DNA was most likely a true copy of the virus genome. The cloning opened the way to manufacturing HBV DNA, HBsAg and HBcAg in almost unlimited amounts using gene technology, instead of painstakingly extracting it from the scarce and highly-infectious patient material. This was important, since these three materials would be used soon in large amounts for diagnostics and vaccine development.

*Final proof* that the Dane particle is the HBV in the sense of Robert Koch’s postulates came from an elegant animal experiment conducted by the German researchers Friedrich Deinhardt (1926–1992), Heinz Schaller (1932–2010) and their colleagues published 1982 in Nature with Hans Will as first author [25]. While it was still not possible to reliably infect cell cultures with HBV, experimental injection of *cloned* HBV DNA into livers of chimpanzees initiated highly efficient replication of HBV and even acute hepatitis B.

## Serological diagnosis of HBV infections

The introduction of Ausria-125 was the beginning of an impressive development in virus diagnostics; however the test had one major disadvantage: the radioactivity caused significant difficulties in the normal diagnostic laboratory. It was therefore a big step forward when it became possible to label the antibodies used with enzymes, and later with chemiluminescence-generating groups. The test principle of the solid-phase sandwich immunoassays has been maintained to the present, however, for assay of numerous antigens and antibodies in many fields of biomedicine, even if the forms of the solid-phase and the signal generation have changed.

**Figure 5:**
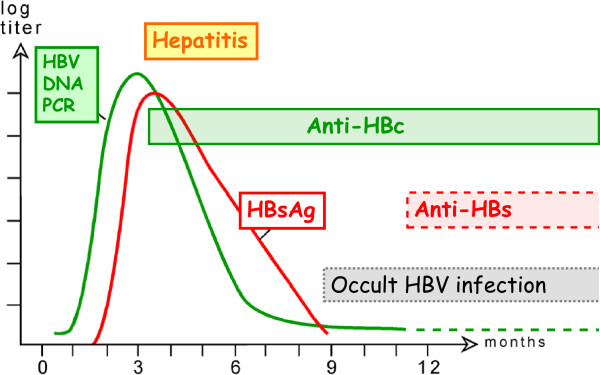
**Schematic representation of the course of acute HBV infections with resolution.** After the infecting event (time 0) follows a lag phase of several weeks without detectable markers. Thereafter HBV DNA (within the virus) and HBsAg increase exponentially in the serum. HBV DNA is detected earlier because its assay is much more sensitive. The peak of HBV DNA and HBsAg is reached before outbreak of the acute disease and both decrease after the onset of clinical symptoms. Initially, the HBV DNA decreases faster because it has a shorter half life time in serum than HBsAg. HBsAg finally disappears whereas HBV DNA may remain detectable in traces. Antibodies against the HBV core antigen (anti-HBc) appear with the onset of symptoms, the protective antibody against HBsAg (anti-HBs) appears very late, usually several weeks or months after disappearance of HBsAg. Disappearance of HBsAg is considered to be a sign of resolution but the virus often remains in occult form in the liver.

The assay of HBsAg was soon complemented by the detection of *antibodies* against HBsAg (anti-HB**s**) and HBcAg (anti-HB**c**). Typically, *anti-HB****c*** appears with the onset of acute hepatitis or after an unnoticed clinically silent HBV infection event. If HBsAg is found without anti-HBc, the patient can still develop hepatitis. If the HBV infection is completely under immune control, the HBsAg disappears, but the anti-HBc remains and *anti-HB****s*** usually appears as a sign of immunity. If the infection becomes chronic, HBsAg and anti-HBc remain positive (Figure 5). With these three HBV markers, it was possible to establish the infection or immunity status of a person in routine diagnostics since the early 1980s.

### Significance of anti-HBs

The very high anti-HBs concentration necessary for a positive reaction with HBsAg in the Ouchterlony technique (as used by Blumberg) was an exception occurring only after repeated exposure to HBsAg. This happened before 1972 in frequently transfused persons as in the case of hemophiliacs. For detection of naturally acquired anti-HBs after HBV infection, the Ouchterlony or other antibody tests such as CFR were not sufficiently sensitive, and a feasible HBV neutralization test was not available. Abbott put the first adequate test for anti-HBs on the market in 1975, which was still a solid phase RIA with Iodine 125-labeled-HBsAg as a reagent. The test was hugely important for the development and licensing of the hepatitis B vaccine, the origins of which went back to a patent of Blumberg in 1970 [1], page 134–146. According to his idea, the first generation of HB vaccines contained purified HBsAg 20 nm-particles from HBV carriers. Successful immunization was and is proven by the detectable presence of anti-HBs. Furthermore, complete recovery from acute or chronic hepatitis B is best demonstrated by a positive anti-HBs result.

### Significance of anti-HBc

The landmark result of Almeida on the development of anti-HBc during acute hepatitis B was soon confirmed by others, in particular by Jay Hoofnagle [26]. The first experimental tests in the 1970s, including CFR, used HBcAg from infected liver or from Dane particles as antigen, but later “recombinant” HBcAg produced in *E. coli*[23] or yeast was used. In the author’s experience, the natural HBcAg yielded highly specific results, but the results obtained with the more readily available recombinant HBcAg from *E. coli* suffered from a certain degree of non-specificity which remains a problem to the present. In 1982, the first commercial anti-HBc test was released as an enzyme immunoassay. Anti-HBc had, in years following, transiently gained significance as a surrogate test in blood donors for hepatitis C infection which could not yet be diagnosed, and also - transiently - for HIV, because of the partially overlapping transmission pathways of HBV, HCV and HIV.

Since anti-HBc neither proves active infection nor immunity, it could be considered clinically unnecessary except for epidemiological studies or confirmatory testing. However, anti-HBc does not only provide evidence of prior infections, but also of an ongoing, occult HBV infection, whereby the word “occult” refers to the apparent lack of HBsAg, which is present in an undetectable amount in such cases. In blood transfusion, up to 200 mL blood plasma is transferred, and the smallest traces of HBV can lead to infection in the recipient. Anti-HBc can be a marker for occult infection as was recognized by Hoofnagle already in the 1970s [27]. For a long time the small residual risk caused by occult infected blood donors was tolerated. But with todays increased safety demands, blood donors have to be tested for both HBsAg and anti-HBc in many countries, e.g. in Germany, since 2006. An anti-HBc determination is also important *before* medically induced immunosuppression, since an occult HBV infection can be reactivated under these circumstances with severe or even fatal consequences. This was already recognized in 1975 by Arie Zuckerman and colleagues [28] but even today not all hematologists are aware of this problem. Reactivation can be suppressed with preemptive antiviral therapy if the problem is recognized in advance.

### Significance of IgM antibodies to HBcAg

Anti-HBc total antibody assays were useful to determine whether a patient ever had contact to HBV but this marker could not distinguish whether the infection was acute or persistent or resolved. It had been long known that the early immune response against an infectious agent induced antibodies of the immunoglobulin class M (IgM, M for macroglobulin), whereas weeks or months later the antibodies belonged mainly to immunoglobulin class G (IgG, G for Gamma). It was therefore only necessary to determine the immunoglobulin class M of the antibodies to distinguish between a fresh and an old infection. This used to be costly and laborious, as biophysical methods (like ultracentrifugation, ion exchange or gel chromatography) were needed to separate IgG and IgM. It was a break-through for virus diagnostics when a very simple test principle was developed for IgM Anti-HAV by Bertram Flehmig (Tübingen, Germany) [29] and independently by the author of the present paper for IgM Anti-HBc [30]: covering the solid phase with an antibody against IgM (anti-μ chain) and consequently using it to capture the IgM from the sample. The HAV-Ag or HBcAg is then added and, if bound by specific IgM, detected with a labeled antibody. Today, this test principle is standard for assay of most antiviral IgM antibodies.

### Quantitation of IgM anti-HBc

Differentiating between an acute and chronic hepatitis B infection was often difficult. Large clinical studies initiated by Reiner Thomssen (Göttingen, Germany) in the 1970s showed that the interpretation of the IgM anti-HBc results obtained by the author was not as straightforward as most clinical virologists had expected. Since the anti-μ capture assay is very sensitive, some patients who resolved acute HB remained positive for years and even patients who had chronic HB without known acute phase were positive. Only an accurate well standardized quantitation allowed distinction of clinically apparent acute from chronic HB [31]. Not all clinical virologists liked this differentiated evaluation and made the test artificially insensitive leading to the problem that mild acute infections were no longer recognized.

One reason why IgM anti-HBc remains moderately positive in chronic HBV infections was detected by David Milich (La Jolla, California). HBcAg is an unusual T cell-independent antigen which can activate B cells to produce IgM anti-HBc [32]. The strong B cell immunogenicity of HBcAg may be part of the immune evasion strategy of HBV because it may interfere with the activation of HBcAg specific cytotoxic T cell reactions. The IgG anti-HBc found in resolved or inactive cases is induced by the normal T cell-dependent class switch.

## HBeAg and immune control of HBV infection

### Discovery of HBeAg

Many researchers, including Blumberg, realized soon that the mere presence of HBsAg does not allow an assessment of the severity of the disease or the infectiousness of the patient, both of which may independently vary within a large range. Some HBV carriers could be very infectious without suffering from an obvious disease. In 1972, Swedish virologist Lars Magnius discovered, while looking for HBsAg subtypes, an additional marker, HBeAg, in HBsAg positive sera which helped to distinguish highly infectious from less infectious HBV carriers [33]. The “e” is not an abbreviation for “early” as some people believe; it stands on its own. According to Magnius its meaning should not be disclosed and considered an ***e****nigma*. For several years, the biochemical nature of HBeAg was also an enigma, but with the advent of artificial expression systems for HBV genes, William Rutter’s team [34] and the author together with Volker Bruss (at that time in Göttingen, Germany), [35] independently showed that HBeAg is a non-particular secreted form of HBcAg. The completely different processing and function of HBeAg is caused by a preC signal sequence for secretion preceding the HBc gene (see Figure 4).

### HBeAg positive HBV carriers

**Figure 6:**
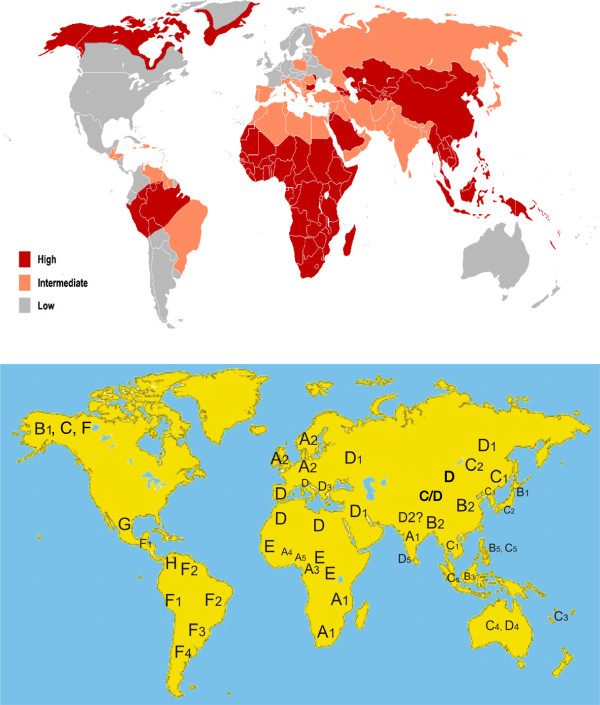
The three phases of chronic HBV infections.

HBeAg is not essential for virus replication. HBeAg negative variants of HBV with a mutated, non-functional preC sequence were first described in 1989 by William Carman [36] and can even cause fulminant hepatitis B. As shown by David Milich, HBeAg acts as an immune modulator and suppresses the recognition of HBcAg expressing cells by T-lymphocytes [37] which is the main mechanism of HBV immune control. The long term lack of effective immune defenses allows production and secretion of the virus with up to 10^10^ infectious particles per mL blood, without the infected person showing clinical symptoms. HBsAg is usually present at very high levels of between 30,000 and 200,000 ng/mL serum (Figure 6).

### Waning HBeAg and chronic hepatitis B

In patients with chronic hepatitis B, the immune defense is partially active, such that the viral loads and HBsAg levels are lower (Figure 6). While the destruction of the HBV-infected liver cells leads to chronic liver inflammation, it does not stop the infection, as new cells are continuously infected in the absence of neutralizing antibodies. Only once the immune defenses become more efficient can the patient reach a condition where HBsAg is still produced, but the production of virus particles is so slight (viremia <10^4^/mL), that it can no longer cause any major damage. These quasi-healthy HBsAg carriers have less HBsAg in the blood (mostly <3,000 ng/mL) and no longer have HBeAg, but rather the corresponding antibody, anti-HBe. A seroconversion of HBeAg to anti-HBe and a significant decrease of HBsAg are considered a good sign for a spontaneous or therapy-induced improvement. But many patients who have lost HBeAg may still suffer from progression of chronic hepatitis B because the virus has an enormous capability to evade T and B cell immunity even if HBeAg is no longer present.

## Measurement of HBV infectivity

### Obstacles

HBeAg gives a hint on the activity of HBV replication and the almost absent host’s immune control. But a more dependable marker for the activity of the infection (not inflammation and disease!) is the level of viremia. Normally, the gold standard for measuring the number of infectious viruses is replication in susceptible cells. Unfortunately, HBV is highly species- and organ-specific. *Permanent* cell lines of human hepatic origin have no or very low *susceptibility* for HBV and even *primary, differentiated* hepatocyte cultures obtained from surgically excised human liver pieces are suboptimal and require inoculation of one hundred thousand or more virus particles for establishment of a detectable transient infection. In contrast, HBV is extremely infectious for humans if it enters the blood stream as has been observed in many outbreaks of the disease. Lewellys Barker and Roderick Murray studied the infectivity of plasma from an acute hepatitis B case systematically by injecting 1 ml dilutions of this plasma to so-called *“volunteers”* (prison inmates) in 1951–54. They found that dilutions of 1:10^4^ still could cause clinical hepatitis. When they re-analyzed their study samples in 1970 with the then available AuAg test they found that dilutions up to 1:10^7^ caused a clinically silent HBV infection [5].

### Animal experiments

Similarly susceptible is the chimpanzee, which was introduced as an experimental animal for HBV research by James Maynard and Robert Purcell in the early 1970s [38] when Krugman’s (in-)human experiments had already raised vigorous public protest. As shown by several groups, highly infectious sera could be diluted by factors of 1:10^8^ and were still infectious in doses of 1 ml when given intravenously to chimpanzees.

Alternatives for highly sensitive *in vivo* detection of infectious HBV are various immunodeficient mouse strains which carry transplanted human hepatocytes, e.g. those developed by Charles Rogler (New York), Jörg Petersen and Maura Dandri (Hamburg) in 1998 [39]. These partially humanized mice are at least as susceptible for HBV infection as the chimpanzees. Since their generation also depends on the very scarce pieces of healthy human or chimpanzee liver, less limited resources are required.

### Cell cultures

As Chinese researchers found in the 1990s, an unprotected small animal species, the Tupaia (*Tupaia belangeri*) living in South East Asia was susceptible for HBV. This squirrel-like animal may be considered a predecessor of primordial primates, the evolution of which branched off as early as 80 million years ago from the line leading to primates. The animal itself develops only a weak infection, but Josef Köck and Michael Nassal (Freiburg) could show that primary hepatocyte cultures from these animals are at least as susceptible for HBV as primary human hepatocyte cultures [40]. Primary Tupaia hepatocyte cultures have been useful for many experimental studies including those of Dieter Glebe (Giessen, Germany) studying the attachment of HBV with the aid of these cell cultures [41].

Re-differentiation of hepatoma cell lines is another possibility to increase the susceptibility to HBV as was shown by Philippe Gripon (Rennes, France) and Stephan Urban (Heidelberg, Germany), and helped to characterize the attachment site of HBV [42]. Since the HBV receptor has been identified recently, hepatic cell lines can be made susceptible by transfecting them with the receptor gene (see below).

### Quantitative measurement of HBV DNA

The assays for HBV infectivity described above were indispensable for HBV research but are too expensive and laborious for the laboratory diagnosis of HB viremia. The best surrogate test is the sensitive and quantitative determination of the number of HBV DNA molecules in plasma or serum. In the early days, the endogenous DNA polymerase reaction developed by Robinson was the first feasible but relatively insensitive and laborious assay. Later this was replaced by various techniques of nucleic acid hybridization (e.g. dot blot) using cloned HBV DNA as labeled probes. These techniques were still of insufficient sensitivity and accuracy and could not detect the low viremia of healthy HBsAg carriers or occult HBV infections. They were, however, useful for distinction of high and low infectivity and for HBV monitoring in early therapy studies.

With the advent of PCR, a new era of medical microbiology began and this has also shaped the hepatitis virology. First approaches in the early 1990s with this method were plagued by many technical problems: cross contamination, inhibitors, false choice of primers and inappropriate extraction of nucleic acids led to almost disastrous results and much initial distrust in this revolutionary method. An international quality control trial from 1995 revealed that only 10 among 39 participating laboratories were able to deliver faultless *qualitative* PCR results for a panel of coded HBV DNA samples [43] and only the two reference laboratories reached in addition the full test sensitivity. One of the problems encountered by many participants was incomplete DNA extraction. As was shown by the author in 1979 (when he was on visit in Robinson’s lab) the virion-associated form of HBV DNA is covalently bound to a protein [44]. This causes heavy losses of viral DNA unless the protein is removed by protease digestion before extraction. Nowadays, real time PCR for HBV DNA has reached an excellent level of performance with a detection limit close to the theoretical minimum of 1 DNA molecule per reaction mix and a huge dynamic range up to 10^7^ or more. However, comparability of quantitative results obtained with different commercially available test kits was and is a problem. Thus, in 1991 the WHO introduced International Standard preparations and an arbitrary International Unit (IU) of HBV DNA [45]. The number of molecules per IU depends on the assay; but typically 5 molecules correspond to one IU HBV DNA.

## HBV infectivity in real life

### Intravenous inoculation

How does the number of HBV DNA molecules correlate with the number of infectious and replication competent viruses, and how does this translate to infectivity and disease development in real life? Comparisons of the chimpanzee infectious doses and the number of HBV DNA molecules in HBeAg positive plasmas showed that ten or less virus particles are sufficient to start a readily detectable HBV infection if they are *injected intravenously*. These findings are confirmed by observations on the very rare inadvertent transmissions of HBV from blood donors in the early phase of infection when HBsAg and even HBV DNA are not yet detectable by the most sensitive techniques.

### Transmission by close contact

Intravenous injections occur only in medicine or illegal drug use and here contaminations with miniscule traces of infectious blood may transmit HBV, e.g. by re-use of syringes. With the general introduction of single use devices for most invasive procedures transmissions of HBV have become rare. But incorrect procedures leading to hepatitis B outbreaks still occur, in particular during blood glucose testing and many other medical procedures. In normal life, small wounds and intimate mucocutaneous contact may allow transmission from a highly viremic person to others. Epidemiological experience shows that this danger is high when the values exceed 10^7^ viruses/mL plasma, but it is nearly non-existent when lower than 10^5^/ml.

### Variable infectivity of HBV particles

The clear correlation between HBV DNA molecule number and infectious dose is lost if the sample comes from the late or HBeAg negative phase of infection. Using mice with implanted human liver cells as infection system, Junko Tanaka (Hiroshima) and her team could show that in the early phase of an experimental HBV infection in chimpanzees nearly all virus particles were infectious whereas in the phase of decreasing viremia one 50% infectious dose (ID50) contained 100 virus particles [46]. Very recent evaluations of inadvertent HBV transmissions from occult infected blood donors showed that one ID 50 contained as many as 1000 virus particles [47]. Thus, the HBV DNA level is a direct marker for infectivity only if the sample comes from the early phase of infection or from a quasi-immunotolerant carrier with very high viremia. In the other phases of infection protective antibodies may cover up the essential attachment sites or unknown mechanisms favor the generation of mutants with impaired infectivity. The low infectivity of most inactive HBsAg carriers is not only due to the fact that they have relatively low HBV DNA levels (typically below 10^4^ IU/mL) but their HBV DNA is enclosed in virus particles the majority of which is not infectious. Furthermore, low doses of infectious HBV favor an inapparent course of the infection [5]. This is, however, only true if the infected person is fully immunocompetent. Otherwise, the infection may become chronic in spite of a low ID50. If HBeAg negative variants are transmitted to transiently immunocompromised patients they may even develop fatal hepatitis [47].

### HBV transmission to and from health care workers

In the past, the risk of acquiring an HBV infection by performing exposure-prone procedures was so high that after several decades of professional activity (even in low-prevalence countries) the majority of health care workers showed markers of previous or ongoing HBV infection. Thus, many physicians became victims of their professional activities, were highly infectious HBV carriers and thereafter a threat for the patients on whom they performed exposure prone procedures. Since the 1970s, there have been numerous reports on HBV transmissions from health care workers with high viremia to patients, usually during surgery. Most critical were thorax, gynecological and oral surgery. The medical community was sluggish to draw the necessary consequences. Initially, the supervising authorities recommended only that HBV carriers should wear double gloves while doing surgery and be particularly cautious.

### Restrictions for HBV positive health care workers

Only after numerous fatal transmissions and hundreds of infections originating from HBV positive physicians were identified and made public were more restrictive measures taken as suggested in a consensus conference [48]. One measure was to implement quasi-compulsory vaccination for health care workers, another to wear protectice devices e.g. face masks or protective glasses during activities generating sprays like in dentistry. As a consequence, the situation has much improved; e.g. in Germany where approximately 50% of all health care workers were HBV marker positive in 1978, in the year 2000 only 5.5% were positive, i.e. less than the 7.0% found in the average population. The other essential change was obligatory control of HBsAg and anti-HBs in medical staff and the exclusion of potentially infectious health care workers with more than 20,000 IU/ml HBV DNA from exposure-prone activities. In Germany and possibly in other countries, however, after the year 2000 there was an over-reaction whereby almost ***all*** HBsAg positive physicians irrespective of viremia were excluded from any activities with patients including injections or taking biopsies. Since the great majority of HBsAg positive health care workers has spontaneously low viremia or can be treated to achieve low viremia, the restrictions which would have been necessary in previous decades are no longer justified. However, after the failures of the medical community in the past, the responsible authorities often decide on this sensitive matter now in an overly formal manner.

## Variability and pathogenicity of HBV

### Human HBV genotypes

**Figure 7:**
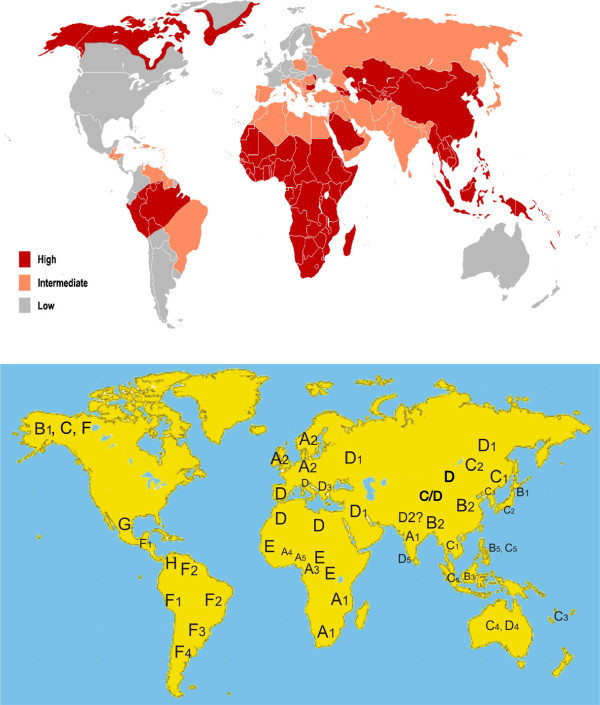
**Prevalence (top) and genotype distribution (bottom) of HBV infections.** Please note that HBV subgenotype A2, present in the most popular hepatitis B vaccines, is only prevalent in the low endemic regions of the Americas and Europe. This means that >99% of all HBV carriers have other HBV subgenotypes.

Besides the already mentioned HBs subtype determinants *d* or *y* and *w* or *r*, Anne Marie Courouce (Paris) found sub-determinants *w1* to *w4* with a typical geographic and ethnic distribution. HBsAg subtype *adw2* was e.g. predominantly found in the USA and northern Europe, *ayw2* in the Mediterranean, *adw4* in indigenous Americans from Brazil and *adr* in East Asia [49]. The immunological subtyping of HBsAg was later extended by sequencing of the HBs gene and eventually the entire HBV genome. Comparisons of HBV DNA sequences from virus strains collected worldwide, performed in 1988 by Hiroaki Okamoto and colleagues suggested the existence of four genotypic groups A - D (later called genotypes) that were divergent by more than 8% in the DNA sequence [50]. This concept was extended by Helene Norder and Lars Magnius (Stockholm) in 2004 to genotypes A - F and the introduction of subgenotypes being divergent by more than 4%. Some of the genotypes such as D were found worldwide whereas others were restricted to one continent like B and C in Asia, E in Africa, and F and H in the Americas (Figure 7) [51]. Genotype E seems to have spread within the human population quite recently because the descendants of those black Americans who were imported 200–400 years ago to the USA from Africa usually do not have this genotype but rather have subgenotype A2 like the descendants of Northern European immigrants [52]. In contrast, subgenotype A1 which is prevalent in Brazil was obviously imported with the slaves from East Africa and also spread via Somalia to the Asian coast of the Indian Ocean [53]. In seeming agreement with these observations, some estimates using bioinformatic algorithms suggested that human HBV has a high mutation rate and may not be older than a few hundred years. But human HBV is probably much older than indicated by these estimates or historic migrations. The existence of a particular subgenotype C4 in Australian aborigines but nowhere else in South East Asia or Oceania suggests that genotype C existed before modern man reached Australia approximately 50,000 years ago and has there independently evolved from the original genotype C ancestor [54]. The evolution of HBV subgenotypes during prehistoric migrations within the last 15,000 years has been recently confirmed by more refined bioinformatic analyses [55]. In early 2013, genotypes A - H (or putative I) and subgenotypes A1 - 7, B1- 9, C1 - 16, D1 - 9 and F1 - 4 have been identified. But the grouping, e.g. of C subgenotypes [56] is under debate because some of the (sub)genotypes, including I, may in fact be recombinants of other (sub)genotypes. HBV genotypes are not only interesting for anthropology and epidemiology but are also useful for clinical reasons. Genotypes C, D and F are on the average more pathogenic than the other genotypes and genotypes A and B respond better to an interferon therapy than genotypes C and D.

*Primate HBV.* HBV as a virus species has a narrow host range but is not restricted to humans. Own variants of HBV have been detected in chimpanzees, gorillas, orangutans and gibbons, and these variants are no more divergent from the human genotypes A - H than the human genotypes among themselves [57]. In contrast, the HBV-like virus of the New World woolly monkey detected by Robert Lanford is much more divergent, not as infectious for chimpanzees as the human genotypes and, thus, considered an individual virus species [58]. These phylogenetic relationships may suggest that HBV-like viruses have existed in primates even before the branching-off of the New World monkeys more than 26 million years ago. Today’s HBV may have existed already when the hominini (ancestors of modern homo sapiens) branched off 6 million years ago from the chimpanzees. Observations in other animal species (see below) suggest that HBV-like viruses have indeed existed for at least 80 million years. Unlike HIV, there is no evidence that human HBV strains originate from primates or vice versa but it cannot be excluded that cross-species from unknown sources have occurred (see below).

### Low variability during the immunotolerant phase

Compared to HIV or hepatitis C virus, the HBV genome sequence is very stable in most cases of acute or chronic hepatitis B. Its sequence usually remains unchanged when HBV is transmitted from an HBeAg positive source to a non-immune person. According to the author’s experience, the subgenotype A2 strains from Central European patients have virtually all the same sequence with only very minor changes [59]. This high genetic stability of HBV is partially the result of the extremely efficient usage of the short HBV genome resulting in overlapping reading frames and numerous regulatory, replicative or morphogenetic elements within the reading frames all of which restrict formation of viable mutations. While the reverse transcriptase of HBV is highly inaccurate, a kind of quality control is achieved by the fact that only successfully transcribed and processed genomes, i.e. partially double-stranded DNA are enclosed in the secreted virus. Another, possibly as important factor is its survival strategy which largely depends on immune tolerance against the structural virus proteins. Immune tolerance is only maintained when the antigen does not change and this is the case in highly viremic long term HBV carriers. Tolerance against HBcAg is induced and maintained by HBeAg, and possibly by the interfering non-protective B cell reaction to HBcAg. High dose immune tolerance against the viral envelope HBsAg may be induced by the huge amounts of subviral HBsAg particles. The expression in the liver (an immunoprivileged organ) and the circulation in the blood in the absence of danger signals also favor development of immune tolerance.

### Pathogenesis of acute hepatitis B

Highly replicative HBV infection is the normal course for several weeks or months before immune recognition begins, after considerable delay. A vigorous cellular immune response suppresses viral replication and eliminates most of the HBV expressing hepatocytes resulting in acute hepatitis. This model of immunopathogenesis has been well supported by studies from Frank Chisari and others in transgenic mice or chimpanzees and by careful analysis of human T cell responses [60]. If the infectious dose is low (typically less than 1000 ID50) the immune response may start early enough before many hepatocytes are infected and in this case the symptoms are so mild that they are usually not noticed [5]. Appearance of neutralizing anti-HBs antibodies in the late acute phase prevents the infection of new hepatocytes.

### Occult HBV infection

Elimination of HBV genomes is usually not complete after acute hepatitis B (even after mild infections) because some HBV genomes remain as cccDNA in an occult form in the liver; but their expression is largely controlled by the immune system. The levels of HBV production are in most cases so low that even with the most sensitive techniques no HBV DNA is detectable in serum.

The term occult HBV infection is often used in a misleading form, because according to the usual nomenclature it is used if HBV DNA is positive but HBsAg negative. In the author’s understanding of normal language, the infection is no longer occult if a well-defined infection marker like HBV DNA is detected in the serum. A truly occult HBV infection has to be assumed if anti-HBc with or without anti-HBs is specifically detected irrespective of a negative result for HBV DNA in plasma. HBV DNA is more often detectable if anti-HBs is missing. This is often the case in HCV- or HIV-co-infected persons [61]. As long as the infection remains truly occult, i.e. HBV DNA and HBsAg are negative and HBV expression is low, no pathogenicity is to be expected. The problem is the reactivation under immunodeficiency particularly under medication with B-lymphocyte destroying drugs (e.g. rituximab) which may lead to fatal hepatitis after immune reconstitution [62] or alternatively to highly viremic untreatable chronic infection [63].

### Pathogenesis of chronic hepatitis B

During acute hepatitis B, HBsAg disappears by definition within six months. A longer persistence of HBsAg is considered a marker for chronic HBV infection. Infection of newborns (from the HBV-infected mother) or infants typically results in a persistent infection because for unknown reasons an effective immune response does not begin for years or decades. Infection of immunocompromised patients also leads to persistence even if the immune impairment is mild as in hemodialysis patients. However, after a long anergic phase, immune defense may emerge and lead to selection of escape mutants. As soon as cellular immune responses against HBcAg appear, HBeAg has lost its immunomodulatory function and is a useless side product. HBeAg-negative variants with enhanced HBcAg expression and viral replication usually take over and partially compensate for the loss of destroyed HBV infected cells. Variants with mutated T cell epitopes of HBcAg and HBsAg may be selected, non-essential epitopes of the preS domain may be deleted. The main point is now whether the immune response is strong enough to keep HBV DNA replication low. If so, the course may be benign although expression of HBsAg may still occur (Figure 6). Finally, the immune control will even suppress HBsAg to undetectable levels in many chronic carriers. On the opposite, co-existence of cytotoxic immune responses with ongoing strong HBV DNA replication results in inflammatory disease, progressive fibrosis of the liver and potentially in hepatocellular carcinoma.

### Late phase variability

In this phase of infection (independent of a prior acute, occult or chronic course) the variability of the HBV genome is very high and leads to many defective forms which by themselves may favor increased pathogenicity. Hot spots of variability are the preC and core gene, the HBs antigen loop, and parts of preS. There are several mechanisms of how the preC sequence can be inactivated by a single point mutation. Most common is the introduction of a stop codon instead of a trp codon at the end of the preC sequence, thus preventing translation of the HBeAg precursor. This mutant is more stable in HBV genotype D than in genotype A2. Consequently, HBeAg-negative variants are more prevalent in the Mediterranean than in Central or Northern Europe.

**Figure 8:**
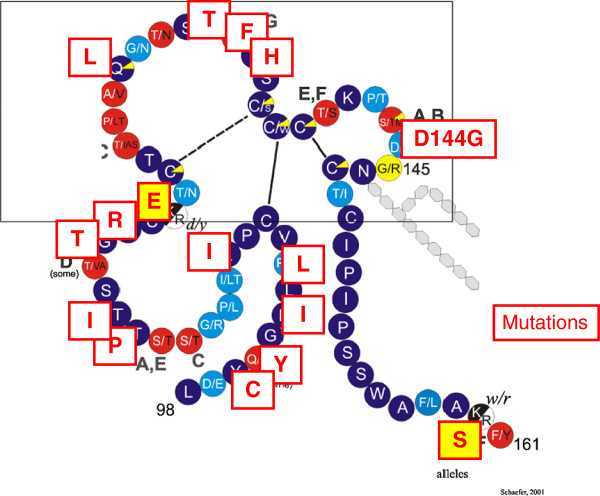
**Mutations in the HBsAg loop of a reactivated HBV variant.** The complicated folded loop forms the surface of HBV and HBsAg particles. The exact topology and three-dimensional shape of the loop are unknown. One circle corresponds to one amino acid in the single letter code of the normal (wildtype) HBV, each square to a mutation. The boxed-in part is named a-determinant and is believed to be immunodominant, but immune escape induced mutations occurred in the entire HBsAg loop. Yellow (shaded) squares cause the loss of an immunodominant HBsAg subtype determinant. This variant replicated in a patient receiving lymphoma therapy. The patient was anti-HBc and anti-HBs positive before the immunosuppressive lymphoma therapy and developed severe acute hepatitis B after end of the therapy due to immunopathogenesis against the variant which had become abundant under immunosuppression. The serum from the acute reactivated hepatitis B phase had a high virus load, but was HBsAg negative in all assays.

The replication via reverse transcription (see below) and the very large number of HBV genomes expressed (up to 10^13^ per patient day) facilitate mutations of every base at least two times per day. In addition, the RNA editing cytidine deaminase APOBEC3G (an interferon induced innate defense factor against retroviruses) may hypermutate the HBV pregenome [64]. Since the immune selection criteria are the same in occult HBV infection, virus strains reactivated under immunodeficiency are often highly mutated, e.g. as described in ref. [62] and [63]. An unpublished example from the author’s laboratory is shown in Figure 8. These highly mutated variants are usually not transmitted because viremia is in these cases much lower than in early or immune tolerant phases. If these variants are, however, transmitted they induce either inapparent infection because of reduced fitness or very severe and even fatal hepatitis B because these variants lack the immunomodulatory potential of wild type HBV [47].

## Oncogenicity of HBV

Blumberg [1], page 147–158 and independently Wolf Szmuness (New York Blood Center) [65] noted already in the 1970s that chronic HBV infection was associated with an increased incidence of hepatocellular carcinoma (HCC). In areas with very high prevalence of chronic hepatitis B, HCC was the most frequent form of cancer. R. Palmer Beasley found in a large prospective study with 22,707 middle-aged men in Taiwan an HCC incidence of 1158 in 100,000 man-years among the HBsAg carriers but only 5 in 100,000 for HBsAg negative persons. In 54% of the HBsAg carriers HCC and liver cirrhosis were the cause of death [66]. Similar situations are found in other regions highly endemic for HBV. HCC enhancing cofactors are co-infection with hepatitis C virus and exposure to aflatoxin.

### HBV DNA in hepatoma cells

While the immunopathogenesis of HBV is relatively well understood, the mechanisms of oncogenicity are not. Early hopes that explantation and cultivation of hepatoma cells from HBV carriers would lead to a culture system for HBV were not fulfilled because the cells usually did not express HBV antigens or HBV DNA. However, in 1976 South-African researcher Jennifer Alexander was able to establish the hepatoma cell line PLC PRF 5 (“*The Alexander cell line”*) which secreted HBsAg [67]. Shortly thereafter the team of William Rutter identified several fragments of HBV DNA integrated at several chromosomal sites in that cell line [68].

### Oncogenic products of HBV

As was shown by several groups, in particular by Peter-Hans Hofschneider (Munich, 1929–2004) and coworkers, HBV-related HCC tissue is derived from clonal expansion of single cells with one or more chromosomal insertions of truncated or rearranged HBV DNA. Insertions of replication competent HBV genomes were never observed. Some of the integrated HBV DNA elements encoded truncated preS/S proteins that had oncogenic potential *in vitro* or in immunodeficient mice [69]. These proteins and the HBV X protein are transcriptional transactivators that may activate deregulated cell proliferation and tumor formation [70]. The role of the X protein was not understood for a long time, because it was not essential for HBV replication in permanent cell cultures. As shown by Ulrike Protzer (Munich) and Massimo Levrero (Rome) and their coworkers, it is however an essential transcription activator for expression of the HBV proteins in differentiated hepatocytes [71].

### Insertional mutagenesis

Besides these directly oncogenic products, insertion of HBV DNA promoters and enhancers may activate cellular oncogenes like myc (see below) or disrupt negative regulators of proliferation. In some cases fusion proteins of cellular growth factors and HBV proteins were even observed [72]. However, there is no typical insertion site or conserved altered cellular factor in human HCC. In fact, integration seems to be a normal event during chronic HBV-infection and leads to clonal growth of seemingly normal hepatocytes, a small proportion of which may finally turn to frank malignancy [73].

### Persistent replication

According to clinico-epidemiological studies the main driving force for development of HCC seems to be continuous HBV replication, particularly in HBeAg negative patients. High HBV DNA levels >10^6^ copies per ml serum are 11 times more often associated with progression to HCC than levels <300 copies/ml [74]. The role of HBV DNA replication in this process is not clear. Possibly, the peculiar structure of the HBV DNA (see below) may by itself have an enhancing effect on DNA repair in already too heavily damaged cells. Successful antiviral therapy can stop chronic hepatitis B and the progression of liver cirrhosis but a slightly elevated risk of HCC remains.

## Non-primate animal models of hepatitis B

### Orthohepadnaviruses

The frequent natural occurrence of HCC in woodchucks, (a marmot-like animal living at the East coast of the USA) prompted Robert Snyder and Jesse Summers (Philadelphia) in 1978 to search for an HBV-like virus in these animals. The woodchuck hepatitis virus (WHV) discovered by them showed virtually all properties of human HBV, about 60% sequence homology, and even a serological cross reactivity of the HBcAg with WHcAg but no cross reaction between HBsAg and WHsAg [75]. Consequently, Jesse Summers proposed the definition of a new virus species and a new virus family named *hepadnaviridae* according to the organ tropism (hepa for liver) and the nature of the nucleic acid. Soon after, a closely related virus was found in ground squirrels that are indigenous to the West coast of the USA, by Patricia Marion in Robinson’s team, supporting this proposal [76]. Both animals have proven very useful in the study of hepadnaviruses. The very high oncogenicity of WHV is caused by integration of WHV DNA and the insertional activation of a woodchuck specific cellular oncogene N-myc [77].

### Avihepadnaviruses

HCC was also known to occur in Peking ducks in China. Chinese researchers found virus-like particles in duck sera and sent serum samples to Blumberg (ref. 1 page 171). In a follow-up of this discovery, William Mason (Philadelphia) found the duck hepatitis B virus (DHBV) in Peking ducks outside China as well and characterized it as HBV-like. DHBV was also hepatotropic, caused very high viremia without obvious disease, overproduced the viral surface protein and had a similar morphology and genomic organization [78]. The advantage of the duck virus was that it could be propagated in embryonated eggs and in readily available primary duck hepatocyte cultures. There were, however, also distinct differences to the mammalian hepadnaviruses. The surface and core proteins had a different structure, there was seemingly no HBx protein and the overall sequence homology was low. The phylogenetic relationships suggested that the genera *orthohepadnavirus* of mammalians and *avihepadnavirus* of birds could be distinguished. The group of Hans Will (Hamburg, Germany) identified further avian hepadnaviruses in various species of water fowl such as herons, cranes and storks. Studies of these viruses confirmed the usually narrow host range of hepadnaviruses and the conservation of the seemingly non-essential HBV proteins HBx and HBeAg [79]. In contrast to orthohepadnaviruses, the avihepadnaviruses do not cause HCC but they may cause hepatitis when infecting adult birds. (The duck HCC in China is caused by chemical carcinogens). Avihepadnaviruses apparently have existed for >82 million years in birds, because various bird species carry fragments of avihepadnaviral DNA as an endogenous viral element integrated into their genome [80].

Neither ortho- nor avi-hepadnaviruses have economic importance as animal pathogens. The animal viruses were, however essential for the progress in our understanding of HBV infection. DHBV was crucial for the elucidation of the hepadnaviral replication cycle and the WHV-infected woodchuck has become an important animal model for pathogenicity and therapy of human HBV infections.

## Hepadnaviral life cycle

### Unusual structure of HBV DNA

Robinson had described the nucleic acid of HBV as a small circular double-stranded DNA but he found that the HBV DNA was profoundly different from that of other DNA viruses. In contrast to polyoma- or papillomaviruses the circular DNA was not covalently closed but had a nick in one strand and a variable gap in the other [19]. As could be shown by the author in 1980, the 5’ends of the DNA strands were blocked, in case of the longer minus strand by a at that time unidentified protein [44]. All hepadnavirus species have this genome structure (Figure 3). The mechanism by which these structures were generated appeared enigmatic.

### Reverse transcription of pregenomic RNA

The solution of the enigma came soon with the publication of Jesse Summers and William Mason on the replication of DHBV DNA in 1982 [81]. Surprisingly, the DNA polymerase within the DHBV core particles, (isolated from infected duck ***liver***) transcribed in a first step an ***RNA*** template and only in the second step the newly formed ***DNA*** strand as reported by Robinson for ***serum***-derived HBV particles. With this discovery, it became evident that the replication of the *hepadnaviridae* was in some steps similar to that of the *retroviridae.* The similarity to retroviruses is mainly restricted to the ability of the viral DNA polymerase to transcribe both RNA and DNA and to remove by the coupled RNase H activity the transcribed RNA template.

### HBV DNA replication

**Figure 9:**
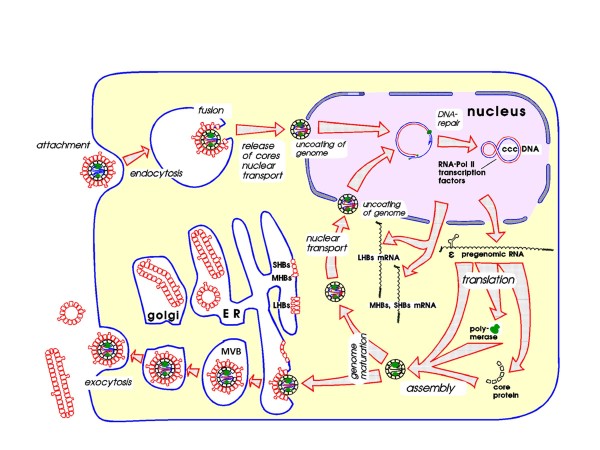
**Life cycle of HBV.** Attachment to liver-specific receptors (heparansulfate proteoglycan and NTCP, see text) leads to endocytosis of HBV and release of HBV core particles. These are transported to the nucleus and arrested at the nuclear pore complex where the HBV genome is released to the nucleus. In the nucleus, the viral DNA is “repaired” to the covalently closed circular (ccc) DNA and complexed with nucleosomes (not shown). In interaction with transcription factors (not shown), the ccc DNA is transcribed to the pregenomic and subgenomic mRNAs. The mRNAs are transported, mainly without splicing, to the cytoplasm. The two subgenomic mRNAs for the three HBs proteins are translated at the endoplasmic reticulum, assemble to subviral HBsAg particles and are secreted via the Golgi apparatus. In parallel, the pregenomic mRNA is translated in the cytosol to the HBV core protein and the viral polymerase, whereby the three components assemble to the immature core particle. The HBV genomes mature within the core particles via reverse transcription of the pregenomic mRNA to DNA. The mature core particles can migrate again to the nuclear pore complex or are enveloped by the surface proteins and secreted via the multivesicular bodies (MVB).

Due to the efforts of many eminent virologists, among them Jesse Summers, Pierre Tiollais, Harold Varmus, Heinz Schaller, Don Ganem, Michael Kann, Dan Loeb, Michael Nassal, Christoph Seeger, John Tavis and their coworkers, the following picture (Figure 9) of the HBV life cycle has taken shape within the last 30 years as described in a review from Michael Nassal (Freiburg, Germany) [82]. After specific attachment to differentiated hepatocytes and endocytosis (see below), the viral envelope is removed, the core particle is actively transported to the nuclear pore and within the nuclear pore the viral DNA is released to the nucleoplasm [83]. There it is converted to covalently closed circular (ccc) DNA by cellular repair factors and remains an episomal minichromosome. The cccDNA binds liver-specific and ubiquitous transcription factors and is the template for the cellular RNA polymerase II which generates the pregenomic and subgenomic mRNAs. The pregenomic RNA is translated to core protein and by internal translation initiation to DNA polymerase. The core protein subunits assemble spontaneously to immature core particles which with the help of cellular chaperones encapsidate their own mRNA and the viral DNA polymerase. Reverse transcription starts only within the core particle at a specific secondary structure of the pregenomic RNA called ε for “encapsidation” because this site, together with the polymerase, also mediates the selective encapsidation of the pregenomic RNA. Hepadnaviruses use a hydroxyl group of a tyrosine residue in their DNA polymerase for priming of their first (minus) DNA strand. This creates the strange covalent linkage between HBV DNA and protein. After completion of the first strand and degradation of the pregenomic RNA by the viral RNase H, the capped 5’terminal remnants of the RNA are used as primer for the plus strand synthesis. As shown by Volker Bruss, only mature core particles which contain a complete DNA minus strand and a partial plus strand interact with the preS domain of the membrane associated viral surface proteins and acquire the viral envelope [84]. Interestingly, the preS domain is initially in the cytosol, but later about half of these preS domains are translocated to the virus surface where they can function as attachment site [85]. For a long time it was believed that HBV is secreted via the Golgi apparatus but this is only true for the small HBsAg particles. Complete HBV was recently shown by Reinhild Prange to be exported via multivesicular bodies like many other enveloped viruses [86]. The very interesting details of this entire process shall not be presented here and are still under investigation in many laboratories.

This replication cycle is very different from that of the *retroviridae*. Retrovirus particles contain their genome as two copies of RNA and start reverse transcription only *after* entry into the target cell. They use tRNA as primer for their minus strand DNA. The completed double stranded DNA is linear and needs to be integrated into the host genome before synthesis of the pregenomic RNA. The progeny virus is assembled and released at the plasma membrane as immature particles which require cleavage of the viral gag proteins by the viral protease to become infectious.

### Identification of the HBV receptors

In 1987, Mary Ann Sells and George Acs succeeded in generating the stably *HBV-transfected* hepatoma cell line HepG2.2.15 which was *permissiv*e for replication of infectious HBV and has been crucial for many studies on HBV, in particular for development of antiviral drugs [87]. But this and similar cell lines did not facilitate the search for the factors mediating efficient *attachment and entry* of HBV. Many publications claimed to have detected functional receptors for HBV, but the search for them remained unsuccessful for decades. Only recently, Camille Sureau (Tours, France) could prove by a meticulous mutational analysis that the previously by J. P. Allain identified *heparansulfate proteoglycan* binding capacity of the small HBsAg protein [88] is essential (but not sufficient) for infectivity and that the binding sites coincide with neutralizing epitopes of HBsAg [89]. However, this receptor cannot explain the peculiar species specificity of HBV because it is present in livers of all mammalians.

The species-specificity of attachment to an uptake mediating receptor rests in the amino terminal preS1 domain of the large viral surface protein. The *large HBsAg protein* is a minor component in the 20 nm HBsAg particles but a main component of the virus particles. It remained unrecognized for many years but was finally identified by the author together with Klaus Heermann in 1984 [90]. Soon after in 1986, Alfred Neurath (New York) characterized the preS1 domain as potential *attachment site* of HBV to hepatic cells [91] but it took another 26 years to identify a functional cellular receptor for this attachment site. Don Ganem (San Francisco) showed in 1995 that the species-specificity of HBV-like viruses in birds was mediated by the preS domains of their large surface proteins [92] and identified later an avian receptor for this attachment protein [93]. The studies from Dieter Glebe on the preS1-dependent infection of Tupaia hepatocytes [94] inspired Li Wenhui and colleagues (Beijing, China) to start a genome-wide search for liver-specific surface molecules common to humans and Tupaias. Very recently, they identified the liver-specific *sodium-dependent taurocholate cotransporting polypeptide* (NTCP) as an essential receptor for the preS1 attachment site of HBV and could prove its specificity by the fact that this receptor from Tupaias and man (a susceptible primate species) but not from non-susceptible primates mediates HBV infectivity [95]. The receptor is only expressed in the intact liver, disappears within a few days in primary hepatocyte cultures and is absent in undifferentiated hepatoma cell cultures.

## Therapy of hepatitis B

In the 20th century, therapy of viral infections lagged far behind the impressive antibiotic therapies against pathogenic bacteria. The *in vitro* cultivation of viruses was and is much more difficult than cultivation of most bacteria. Furthermore, viruses are so closely embedded in the biochemical host cell machinery that it appeared for long almost hopeless to find Paul Ehrlich’s magic bullet (a substance that selectively targets a pathogenic organism, but not the host) against them. An alternative approach was the adoption of natural antiviral defense factors for therapy.

### Interferon

In 1957 Isaacs and Lindenmann reported that the presence of inactivated influenza virus in an infected cell induced production of a secreted cellular factor, called interferon, which protected neighboring cells against infection not only by influenza virus but many other viruses as well. The early hopes to use interferon as a universal drug against all or at least many viruses did not materialize in clinical practice. But in 1976, William Robinson and Thomas Merigan (Stanford, California) reported that interferon alpha (at that time produced in human leukocyte cultures and very expensive) suppressed HBV replication and cured some patients suffering from chronic hepatitis B [96]. Further clinical studies showed that only a minority of the patients could be cured by this therapy while the majority showed a relapse after the end of therapy or even viral breakthrough under therapy.

### Indication for interferon therapy

After 36 years of experience, interferon alpha (meanwhile recombinant and in polyethylene glycol-conjugated form) still has its place in HBV therapy, but the patients need to be carefully selected, because interferon has many severe side effects and contra-indications, and only a minority will show a sustained response. Interferon suppresses HBV replication but the exact mechanism is not known and today more dependable chemical antivirals with less side effects are available. The main advantage of interferon is that it can enhance the body’s own immune defense, and accelerate the *sustained resolution* of the infection. Thus, patients with active inflammation, i.e. elevated transaminsases, and partially successful immune control (i.e. moderate viremia < 10^8^ IU/ml) are the best candidates for this strenuous therapy. The cccDNA form of the HBV genome is probably as stable as the host chromosome and can currently not be attacked by any available drug. However, interferon may induce innate defense mechanisms e.g. the RNA editing cytidine deaminase APOBEC3G, which may damage the HBV pregenome, or it may enhance apoptosis of infected cells. Accelerated hepatic cell turnover in absence of HBV replication will decrease HBV cccDNA to innocuous levels. Long-term follow-up has confirmed that patients with a sustained viral response regain a normal life expectancy [97].

### Monitoring of therapy

Since best results are obtained with 48 weeks of interferon therapy, it is necessary to recognize as early as possible those patients who will not develop a sustained response. The decrease of the HBV DNA level in plasma is a necessary condition but not suitable as predictor for long-term success. The usefulness of monitoring the HBsAg concentration for the early prognosis of acute hepatitis B was reported by the author already in the 1970s [98], but these findings were ignored until recently. In the last years many clinical studies showed that the decrease of the HBsAg level indicates in most cases indirectly the decreasing amount of intrahepatic cccDNA due to immune elimination of infected cells. If the interferon therapy leads to sustained response, a significant decrease of HBsAg is detectable within 12 weeks. An important point to consider is that not all HBV genotypes are equally susceptible to this therapy: genotype A and B are more responsive than C or D [97].

## Inhibitors of reverse transcriptase

Although viruses become parts of their host cell during their life cycle, basic research on their replication mechanisms identified various virus-specific biochemical pathways that could be targets of pharmaceutical intervention. The nucleoside analogue acyclovir against herpes simplex and varicella zoster virus was the first example of a successful nontoxic antiviral drug taylored to the biochemistry of its viral target. Merigan and Robinson turned in the late 1970s to then available inhibitors of DNA polymerases when they realised that interferon alone would not help the majority of patients. However, these early drugs, though effective, were highly toxic, in particular to the mitochondrial DNA polymerase γ. With the advent of HIV in the developed countries, research on antiviral therapy got a strong boost leading to the first drug azidothymidine in 1987 which inhibited more specifically the reverse transcriptase of HIV, but it was unfortunately inactive against HBV.

### Lamivudine

In 1991 Raimond Schinazi’s team reported inhibition of HBV DNA synthesis in HepG2.2.15 cells by the HIV drug lamivudine, a thio-derivative of deoxycytosine [99]. After licensing lamivudine for therapy of HIV it was noted by Yves Benhamou and colleagues that patients who were co-infected with HBV lost (transiently) their HB viremia and showed clinical improvement of hepatitis [100]. The first successful short term clinical trial with lamivudine in HBV monoinfected patients was published in 1995 by Jules Dienstag [101].

Although it appears logical, it is not self-evident that RT inhibitors lead to rapid improvement of HBV associated inflammatory liver disease because they do not inhibit HBV antigen expression. As pointed out above, the current view of HBV induced immune pathogenesis would suggest that the RT inhibitors act slowly by preventing infection of new cells while the still ongoing antigen expression of viral antigen in the infected cells would lead to their immune recognition and elimination. For unknown reasons, the suppressed DNA replication leads directly to a decrease of cell damage with enhanced survival of the already infected cells. Thus, the RT inhibitors cannot eradicate the pre-existing cccDNA and must be given continuously.

Lamivudine was quite effective and well tolerated, but did not turn out to be the solution to the problem. Unfortunately, resistance soon developed in cases with high replication. After 5 years of therapy 75% of the treated patients had resistent HBV strains [97]. Due to the overlapping polymerase and HBsAg reading frame some of the resistance mutations also led to a mutated HBsAg sequence with reduced binding to diagnostic or protective anti-HBs antibodies [102]. With HIV, the resistance problem was rapidly countered by introduction of a triple combination therapy, but for HBV this concept was not immediately feasible. Newer drugs had to be developed. Today, lamivudine is no longer justified as a first line drug against HBV due to the resistance problem and this has to be considered when planning HIV therapy in HBV-coinfected patients. An additional problem is that lamivudine resistant strains are able to acquire resistance to the very efficient drug entecavir which is not prone to resistance if used as first drug. For short term treatment, e.g. of very severe acute hepatitis or for prophylaxis of HBV reactivation Lamivudine may still be an option.

### Adefovir and Tenofovir

The acyclic nucleotide analog adefovir was described as early as 1989 by DeClercq as an inhibitor of various retroviruses in vitro, but it took until 2002 to receive approval for the therapy of lamivudine resistant HBV infections. Unfortunately, its activity on HBV was relatively low and completely absent in about one quarter of the patients. Furthermore, it induced in many cases selection of resistant HBV mutants with a stop codon in HBsAg, reminiscent of the HCC-associated truncated preS/S proteins [103]. In the meantime Adefovir should be completely replaced by newer drugs such as tenofovir. This structurally very similar drug was approved in 2001 as HIV drug and was soon shown to be superior to adefovir for HBV as well [104], but it was only approved for HBV in 2008. Today it is the drug of choice for chronic hepatitis B, because it is well tolerated and resistance has never been observed [97]. However, both adefovir and tenofovir have nephrotoxicity as side effect.

### Entecavir

The guanosine analog entecavir was originally developed against herpes simplex virus but was not adequately effective. In 1997 its very strong activity against HBV was shown in the HepG2.2.15 cell line. In 2001, a first proof of principle short term study showed its efficacy in patients with lamivudine resistant chronic hepatitis B. Official approval followed in 2006 after large clinical studies proved the superiority of entecavir over lamivudine. Resistance development is low in treatment-naïve patients but with pre-existing lamuvidine resistance more than half of the patients develop resistance against entecavir as well. In rare cases of suboptimal tenofovir efficacy a combination with entecavir may be useful [97].

### Other approaches

Several *other RT inhibitors* have been licensed but they have not reached widespread application. *Telbivudin* has a significant resistance problem, but it has been successfully used to suppress high viremia in HBV infected pregnant women before delivery, thus protecting the newborn from an excessive viral load [105]. Together with tenofovir it is the only NIH class B drug for treatment during pregnancy.

In contrast to HIV, combination therapies, e.g. interferon with RT inhibitors have not demonstrated superior results. For prevention of re-infection after liver transplantation in a previous HBV carrier a combination of *RT inhibitors* with hepatitis B immune globulin (*HBIG)* may be useful. The competitive *attachment inhibitor* myrcludex [106], a preS1-lipopeptide developed by Stephan Urban (Heidelberg) is undergoing clinical trials and may be particularly useful against hepatitis delta virus which has adopted the HBV envelope. Therapeutic vaccination with HBsAg in combination with RT inhibitors or immunostimulatory substances has not been successful in clinical studies. Immunotherapy with various HBV-derived antigens or HBV antigen-expressing DNA in combination with antiviral therapy has been undertaken in various experimental systems, e.g. by Michael Roggendorf in infected woodchuck with partial success [107]. Targeted destruction or silencing of the hepadnaviral cccDNA would be the ultimate therapy, but is still science fiction.

## Vaccination

In view of the still unsatisfactory therapy of HBV infections prevention has highest priority. Besides obeying strict hygiene with all invasive procedures and a considerate life style, vaccination is the most important way to prevent hepatitis B diseases.

### Passive immunization

Development of the active vaccination took from Blumberg’s first idea in 1968 to introduction to the market 15 years, but passive immunization was more rapidly available. Human immunoglobulin preparations containing very high levels of anti-HBs (HBIG) have been proven useful in protecting persons even shortly after exposure to HBV, e.g. after a prick with a blood contaminated injection needle. Most urgent was the protection of newborns from HBV infected HBeAg positive mothers because they become in 70 - 90% of the cases chronic HBV carriers. In the early 1980s, R. Palmer Beasley conducted a controlled study in Taiwan showing that intravenous HBIG given immediately after birth could prevent HBV infection in 71% of newborns from HBsAg and HBeAg positive mothers [108]. However, the passively administered anti-HBs fades with a half-life of 22 days and is, if given alone, only an interim solution.

### First generation vaccines

Saul Krugman was the first to report on a so-called vaccination against hepatitis B in 1971. He diluted Australia antigen positive serum from his previous human experiments 1:10, boiled it briefly to kill the virus and injected this material to mentally handicapped children as a kind of vaccine. After two injections, he injected infectious HBV containing serum as challenge to the children and found incomplete, but statistically significant protection [109]. The study suggested that Australia antigen positive serum would contain a protection inducing antigen, but due to the crude nature of the “vaccine” it was not conclusive. Using chimpanzees as experimental animals, Robert Purcell and John Gerin from the NIH, and in parallel Maurice Hilleman from MSD, could prove in 1975 that Blumberg’s concept of using *purified* 20 nm HBsAg particles from carrier plasma as HB vaccine was valid and could protect against an intravenous challenge with 3000 chimpanzee-infectious doses [110]. Residual HBV infectivity of the HBsAg preparation was removed by treatment with formalin. Philippe Maupas and Alain Goudeau went one step further and used purified formalin-treated purified HBsAg as vaccine (later produced by Institute Pasteur, Paris) for staff and patients of hemodialysis wards who at that time had a very high risk of HBV infection. In 1976 they reported good protection rates particularly in the staff [111] but they had only a historical control group and, thus, not all observers acknowledged these findings as convincing. A state of the art field study was published by Wolf Szmuness in 1980 using the plasma-derived vaccine produced at MSD. Szmuness had recognized that male homosexuals in New York had an extremely high incidence of HBV infections and performed a large placebo-controlled study with 1083 (truly voluntary) participants [112]. The protection rate of the vaccine was 92%. After this study, the recommendation to vaccinate all kinds of high risk groups, including medical staff, was adopted in many countries.

However, the newly developed vaccine was not well accepted. Soon after the landmark study of Szmuness it became apparent that a part of the HBsAg carrier plasma used for production of the MSD vaccine came from donors who later developed AIDS. At that time the agent of AIDS was not yet identified and it could not be guaranteed that this unknown agent would be inactivated by the treatments sufficient for HBV infectivity. Furthermore many unspecific fears existed about oncogenicity or autoimmunity caused by the vaccine. The main disadvantage was, however, that with increasing acceptance and success, this type of vaccine would have dried out its own resource, i.e. the chronic HBV carrier.

### Second generation vaccines

With the cloning of the HBV genome and the identification of the HBs gene in 1979 a new era of vaccine production was opened, although the beginning was not straightforward. HBsAg could not be expressed in *E. coli* in spite of initial claims. The open reading frame for HBsAg was identified with the aid of the partial amino acid sequence of the major protein of the plasma-derived HBsAg which had been determined in 1977 by Darrell Petersen and Girish Vyas (San Francisco). They found that the major HBsAg protein existed in an unglycosylated and a single N-glycosylated form with ca. 220 amino acids [113]. The larger proteins found in purified HBsAg (see Figure 2) could be removed by treatment with proteases seemingly without loss of HBs antigenicity. The vaccine from MSD was in fact treated with pepsin and did not contain these larger proteins. Thus, it appeared logical to use the gene encoding the major HBs protein for production of the “recombinant” vaccine, i.e. using recombination of the gene with the DNA of expression vectors. Expression of glycosylated and secreted HBsAg particles in mammalian host cells was possible but the yield was relatively low and mammalian cell culture is relatively expensive. Thus, in the early 1980s Pablo Valenzuela for MSD [114] and Michel de Wilde for Smith Kline RIT (today GSK) [115] generated yeast cell strains which expressed the major HBs protein in very large amounts (800 mg/liter yeast culture) at low cost. The product was not completely identical to the natural HBsAg, because it did not assemble spontaneously to the 20 nm particles, was not secreted and not glycosylated. But protection experiments in chimpanzees published in 1984 by MSD were very encouraging [116] and field studies in newborns from HBsAg and HBeAg positive mothers performed in Thailand by Yong Poovorawan and GSK were also very convincing [117]. Thus, in 1986 yeast-derived HBsAg became the standard vaccine against HBV. Rapid immunization schemes for urgent cases (e.g. 3 injections within 3 weeks) are possible but three or four injections within 6 or 12 months are optimal for induction of dependable long lasting immunity. With the advent of an inexpensive, but highly protective and well tolerated hepatitis B vaccine, WHO recommended in 1992 to implement universal childhood vaccination worldwide and meanwhile ca. 180 countries have adopted this measure [118].

### Medical impact of the vaccine

Taiwan was the first country to begin with universal childhood vaccination in 1984. At that time, the rate of perinatal transmission was extremely high leading to a nation-wide HBsAg carrier rate of ca. 10%. Twenty years later only 1.2% of those borne after beginning of the vaccination campaign were HBsAg carriers. Although HCC is mainly a disease of advancing years, the impact of the vaccination quickly became apparent in children and adolescents because its incidence dropped significantly from 0.57 to 0.17 in 100,000 person years in that age group after the beginning of mass vaccination. Thus, the hepatitis B vaccine was the first successful vaccine against a specific form of cancer [119]. Similar observations were reported from other parts in the world. In low prevalence countries like Italy the vaccination has probably contributed (among other factors) to a very strong decrease of hepatitis B incidence.

Observations in Taiwan [120] and Thailand [121] or in high risk groups of other countries suggest that the protection becomes weaker within 20 years but the immune memory is good enough to mitigate the infection in the ca. 23% of those infected. Those with no or with low anti-HBs are still protected against HBV disease but they get a *clinically silent infection* with transient HBs antigenemia, or anti-HBc seroconversion or increase of the anti-HBs titer. The necessity or timing of later booster injections is a matter of debate.

### Shortcomings of the vaccine

The early studies with the plasma- or yeast-derived vaccines suggested excellent immunogenicity with 99% seroprotection rates in healthy children or adolescents. However, in adults the rate of *unprotected non-responders* (with <10 IU/ml anti-HBs 4 weeks after the last dose) is ca. 5 - 7% [122] and increases to 70% under unfavorable circumstances [123]. Risk factors for non-response are numerous: male gender, old age, obesity, smoking and various situations in which the immune system is impaired, e.g. diabetes or hemodialysis. Most important is the *failure of protection against perinatal transmission* in ca. 10 - 20% of newborns from HBsAg and HBeAg positive mothers because they become chronic carriers with the most negative prognosis. These failures are the reason for a 30% residual incidence of HCC in Taiwan [119]. In ca. 23 - 28% of the perinatal vaccine failures HBsAg *escape mutations* have been observed whereas in the pre-vaccination era the proportion of these mutants was only 8%. Fortunately, they no longer appear to be on the increase and have not (yet?) gained epidemiological significance [124].

The *clinically silent virus breakthroughs* in vaccinated persons may be considered insignificant but these infections may create a problem for the HBV safety of blood [125] or organ donations. Furthermore, it appears that these silent infections may *persist* in occult form in the liver and may *reactivate* with serious consequences if the person becomes immunodeficient [126]. The occult or reactivated HBV strains in most cases contain several HBsAg escape mutants against which the vaccine-induced immune response may not protect as shown in Figure 8. A possibly relevant weakness of the most widespread vaccines is that they represent only the HBV subgenotype A2 while >99% of the HBV carriers worldwide have other genotypes (Figure 7). Observations in American blood donors suggest that protection against the homologous HBV genotype is complete even if the anti-HBs level is low (<100 IU/L), whereas such low levels allow transmission of other HBV genotypes as frequently as in unvaccinated persons. The important benefit of the vaccination is that these heterologous infections remain clinically silent and do not result in frank chronicity [125].

### Other vaccine concepts

Overall the current vaccine is an enormous success, however, with a few obvious improvements nonresponse, viral breakthroughs and occult infections could be minimized. Furthermore, a highly potent vaccine could possibly be the basis for an immune therapy of chronic hepatitis B and high chronic viremia. While a complete elimination of the virus from the organism is probably impossible, a successful therapy would lead to a significant decrease of infectivity in the HBV carriers. Together with a high rate of immune persons, this will finally allow for eradication of HBV from the population as suggested e.g. by Ding Shinn Chen [127]. Decades of experience suggest that the protection induced by the vaccine depends mainly on a sufficient level of neutralizing antibodies and a long-lasting B cell memory. With today’s knowledge of HBV biology, the design of a hepatitis B vaccine would probably be different. The preS1 domain of the large HBs protein is the most important attachment factor and the most effective target of neutralizing antibodies [128]. In contrast to the HBs antigen loop, it is highly conserved in the various HBV genotypes and escape mutants are virtually unknown. The designers of the recombinant vaccines [116,117] knew in 1980 that there was a preS sequence upstream of the HBs gene but since the protease treated HBsAg seemed to have full antigenicity and immunogenicity, they neglected it. Once the preS domain was discovered to be a real viral component and its functions for HBV were understood, several entrepreneurs have developed and tried to introduce *preS-containing vaccines*[129,130], but they are rarely used in spite of superior results in field trials particularly in non-responders to the yeast-derived vaccine [131]. HBsAg with or without preS1 expressed in *mammalian* cell culture would be an additional improvement because it contains only the relevant conformational HBs epitopes and no misfolded antigen [128]. The approach of *DNA vaccination* was in fashion for a while but it turned out to be more suitable for mice than for men. All kinds of *viral vectors* were considered as potential carriers for the HBs and preS gene, e.g. Semliki forest virus with promising preliminary results [132], but none of these have ever entered human trials. After almost 30 years of recombinant hepatitis B vaccines some of their deficits are now quite apparent, but the only recognizable progress in practice is the introduction of stronger adjuvants for certain groups of weak responders. This course is a double edged sword because improved immunogenicity may be paid for with more side effects.

## Perspectives

Looking back on the 50 years since the discovery of Australia antigen the development in viral hepatitis is an exemplary reflection of the progress in medicine as a whole through biomedical science. The level that has been reached is formidable, and the scientific possibilities are almost too great to imagine. There is still much to do, especially in medical practice. Quantitative assays for HBV DNA, HBsAg and anti-HBs should be performed more often and their standardization should be improved. Specificity and sensitivity of anti-HBc assays is still unsatisfactory. The significance of occult HBV infections and the danger of reactivation are too often unknown or neglected. The dimensions of HBV variability and pathogenicity have still to be explored. Indication and monitoring of therapy including well-founded stopping rules should be further optimized. Medications leading to sustained cure should be sought for. Vaccination coverage should be constantly increased, particularly in those populations in which it is most necessary. Current hepatitis B vaccines are quite good compared to other anti-viral vaccines but this does not mean that improvement is unnecessary. An effective immune therapy for HBV would be highly desirable and a great stimulus for research on infections against which not even preventive vaccines are available, e.g. HIV or HCV. With sufficient efforts in diagnosis, prevention and therapy, HBV could be eradicated.

## Competing interests

The author was a consultant to Abbott Laboratories and Novartis Diagnostics and Vaccines before retirement.
